# Chitosan/Lignosulfonate Nanospheres as “Green” Biocide for Controlling the Microbiologically Influenced Corrosion of Carbon Steel

**DOI:** 10.3390/ma13112484

**Published:** 2020-05-29

**Authors:** Pathath Abdul Rasheed, Ravi P. Pandey, Khadeeja A. Jabbar, Ayman Samara, Aboubakr M. Abdullah, Khaled A. Mahmoud

**Affiliations:** 1 Qatar Environment and Energy Research Institute (QEERI), Hamad Bin Khalifa University (HBKU), Qatar Foundation, Doha P.O. Box 34110, Qatar; pabdul.rasheed@gmail.com (P.A.R.); rpandey@hbku.edu.qa (R.P.P.); KAbdulJabbar@hbku.edu.qa (K.A.J.); asamara@hbku.edu.qa (A.S.); 2 Center for Advanced Materials, Qatar University, Doha P.O. Box 2713, Qatar; bakr@qu.edu.qa; 3 Department of Physics & Mathematical Engineering, Faculty of Engineering, Port Said University, Port Said 42523, Egypt

**Keywords:** chitosan, lignosulfonate, sulfate reducing bacteria, biofilm, microbial corrosion, impedance spectroscopy

## Abstract

In this work, uniform cross-linked chitosan/lignosulfonate (CS/LS) nanospheres with an average diameter of 150–200 nm have been successfully used as a novel, environmentally friendly biocide for the inhibition of mixed sulfate-reducing bacteria (SRB) culture, thereby controlling microbiologically influenced corrosion (MIC) on carbon steel. It was found that 500 µg·mL^−1^ of the CS/LS nanospheres can be used efficiently for the inhibition of SRB-induced corrosion up to a maximum of 85% indicated by a two fold increase of charge transfer resistance (*R_ct_*) on the carbon steel coupons. The hydrophilic surface of CS/LS can readily bind to the negatively charged bacterial surfaces and thereby leads to the inactivation or damage of bacterial cells. In addition, the film formation ability of chitosan on the coupon surface may have formed a protective layer to prevent the biofilm formation by hindering the initial bacterial attachment, thus leading to the reduction of corrosion.

## 1. Introduction

Microbiologically influenced corrosion (MIC) of carbon steel is a major cause of metal corrosion and pipeline failure [1,2,3]. It is estimated that MIC accounts for about 20% of the corrosion damage in the oil and gas sector [4]. Despite the tremendous efforts made thus far to improve corrosion management, MIC has remained a pressing issue for the oil/gas sector, where there is exposure of metals to bacteria found in water. Several types of microorganism are responsible for MIC, including sulfate-reducing bacteria (SRB), iron-oxidizing bacteria (IOB), slime-forming bacteria, and iron-reducing bacteria (IRB). Amongst those, SRB are the main microorganisms responsible for MIC by generating sulfide species anaerobically, which causes progressive biocorrosion in the water transport systems [5,6]. The SRB strains produce hydrogen sulfides (H_2_S), metal sulfides, and sulfates as a result of biogenic oxidation/reduction reactions [7,8]. In particular, the production of H_2_S at elevated concentrations creates intrinsic heterogeneity, which accelerates the corrosion process by favoring electrochemical reactions [9,10,11].

Biocorrosion control methods are mainly based on either inhibiting the metabolic/growth activities or altering the corrosive conditions to reduce the adaptation of microorganisms. Different types of approach, such as cathodic protection [12], protective coatings [13], corrosion inhibitors [14], and biocides [15], have been used to control/minimize biocorrosion. Oil/gas industries usually need high concentrations of biocides for water disinfection and controlling SRB biofilm formation thereby reducing biocorrosion [16]. However, the use of conventional biocides may cause harmful impact to the environment since it produces disinfection byproducts in addition to the low efficiency against biofilms, and high operational cost [17]. Different nanomaterials demonstrate strong antimicrobial activities, rendering them potential alternatives to conventional biocides [18,19,20,21,22]. Nanomaterials, such as AgNPs [23], ZnONPs [24], TiO_2_NPs [4], FeNPs [25], graphene [26], CuONPs [27], and metal-nanocomposites [22], have been used for the inhibition of SRB-induced biofilm and subsequent MIC. However, the environmental impact of nanomaterials due to their biological toxicity has restricted their use in practical applications [28,29]. The use of green biocides with lower toxicity, environmentally benign, and ease of use can overcome these issues [30].

Chitosan (CS) is a biodegradable polymer abundant in nature with high hydrophilicity, nontoxicity, antimicrobial properties, and low cost [31]. The antimicrobial activity of CS has been widely established against many microorganisms and it shows a high inhibition rate against both Gram-positive and Gram-negative bacteria [32,33,34,35]. CS also displays anti-biofilm activities with a high ability to damage biofilms formed by microbes [36,37,38]. Due to its cationic nature, CS has been able to penetrate biofilms by disrupting negatively charged cell membranes through electrostatic interaction when microbes settle on the surface [36]. Recently, our research group used ZnO-interlinked chitosan nanoparticles (CZNC-10) as stable biocide formulations against SRBs from industrial waste sludge [24]. The nanoparticles achieved a concentration-dependent SRBs inhibition with over 73% efficiency at 250 μg·mL^−1^. Also, CZNC-10 demonstrated 74% MIC inhibition on carbon steel [39]. The dose of CZNC was limited to 250 µg·mL^−1^ due to the ZnO content in the CZNC biocide considering the environmental toxicity issues. In order to develop a more “green” and efficient chitosan-based biocide, the metal or metal oxide nanoparticles need to be replaced with more environmentally benign alternatives.

Lignin is an abundant natural resource that has been widely used as a potential source for fuel and chemical production [40]. Lignin can be incorporated into different polymeric systems such as dispersants, bioadhesives, biosurfactants, polyurethane foams, and epoxy resins, etc., depending on its solubility and reactivity characteristics [41]. Lignosulfonate (LS) exhibits good water-solubility and anionic characteristic [42]. LS also exhibits antioxidant and antimicrobial properties that extend its potential applications to different fields [43,44]. Both CS and LS are good bactericidal agents, and therefore it is expected that CS-LS complexes can be used as highly efficient and environmentally friendly chitosan-based biocides against SRB induced biocorrosion.

The synthesis of CS-LS polyelectrolyte complexes is mainly based on ionic interaction or ultrasonic homogenization [45,46]. CS cross-linked graphene oxide (GO)/LS composite aerogels have been synthesized by the simple mixing of GO, LS, and CS solutions [47]. The aerogels demonstrated 3D porous structure. These CS-LS hybrids showed non-uniform sizes/shapes and instability above pH 4.5, which restricts their practical applications. To solve these issues, we have introduced a new crosslinking strategy towards the synthesis of stable cross-linked chitosan-lignosulfonate (CS/LS) nanospheres [31]. The optimum composite structure was formed at 1:1 ratio of CS:LS. These 150–200 nm nanospheres demonstrated the highest thermal, mechanical, and bactericidal effect against aerobic *Escherichia coli* (*E. coli*) and *Bacillus subtilis* (*B. subtilis*) bacteria as well as anaerobic SRB. As a proof of concept, 100 mg/L CS/LS-1:1 was able to inhibit SRB growth, as demonstrated by 48.8% lower sulfate reduction [31].

Here, we have investigated the ability of the new “green” CS/LS nanospheres with an optimal 1:1 (CS:LS) ratio to treat SRB induced MIC on SS400 carbon steel. The nature and kinetics of SRB inhibition induced by the CS/LS are thoroughly studied with electrochemical impedance spectroscopy (EIS) and surface characterization methods.

## 2. Materials and Methods

### 2.1. Materials

Chitosan (low molecular weight) with 85% deacetylation (CS), Lignosulfonic acid sodium salt (LS), MgSO_4_, sodium citrate, Absolute ethanol, CH_3_CH_2_OH (≥99.8%), CaSO_4_, NH_4_Cl, NaCl, Na_2_SO_4_, KCl, SrCl_2_, KBr, K_2_HPO_4_, HCl, NaOH, hexamethylenetetramine, sodium lactate, and yeast extract were obtained from Sigma-Aldrich (St. Louis, MO, USA). All analytical grade chemicals were used as received. Carbon steel (SS400) rods of 10 mm diameter were obtained locally. The chemical composition of SS400 is 99.25–100% Fe, 0–0.4% Si, 0–0.26% C, 0–0.05% S, and 0–0.04% P. PhenoCure^TM^ (phenolic resin) was procured from Buehler, Lake Bluff, IL, USA.

### 2.2. Synthesis and Characterization of Cross-Linked CS/LS Nanospheres

CS/LS nanospheres (1:1 CS:LS) were prepared according to our previous work [31]. Briefly, 30 mL of both CS and LS solutions were stirred together at room temperature for 30 min. Then, 450 µL of the cross-linking solution were added gradually, and continued stirring for additional 30 min. The cross-linking agent is a mixture of formaldehyde and sulfuric acid (HCHO/H_2_SO_4_, 40/60 *w*/*w*). The resulting solution was purified by centrifuging at 10,000 rpm followed by washing five times with DI water to obtain CS/LS. The size and morphology of the CS/LS were characterized by FEI Quanta 650 FEG SEM (Hillsboro, OR, USA) and FEI Talos F200X TEM (Hillsboro, OR, USA). X-ray diffractogram (XRD) of CS/LS were measured by D8 Advance (Bruker AXS, Bremen, Germany). The X-ray diffractometer is equipped with Cu-Kα radiation (λ = 1.54056 Å) at 40 kV, 40 mA with a step scan of 0.02° per step and scanning speed of 1° min^−1^. The hydrodynamic radius and Zeta potential were measured by Malvern Zetasizer Ultra (Malvern Panalytical Ltd., Malvern, UK).

### 2.3. Fabrication of Coupons and SRB Culture

The coupons of 8 mm diameter steel bar were fabricated by using SimpliMet 3000 mounting press (Buehler, Lake Bluff, IL, USA) and PhenoCureTM as an outer shell. Then the coupons were polished with EcoMet 2500 Polisher (Buehler, Lake Bluff, IL, USA) in a sequence from 240 to 1200 silicon carbide paper and 6, 3 and 1 µm diamond slurry to get a mirror-like finish. The coupons were cleaned with acetone and sterilized with absolute ethanol followed by storing the dried coupons in a desiccated environment until further use. The surface roughness was analyzed by KLA P17 stylus profiler (Milpitas, CA, USA) and SEM was used the image the surface morphology.

Mixed SRB culture was enriched from biofilm samples from a local oil field in Qatar as described earlier [24]. The SRB was further cultured in Postage’s C medium in a saline media [39,48]. The composition of the inject seawater is given in the Supplementary Table S1. The concentration of SRB biomass was represented by volatile suspended solids (VSS) available in the culture media [24]. The known volume of the SRB biomass were taken and oven dried at inert atmosphere to calculate the VSS. The carbon steel coupons were incubated in 200 mL bottles containing 250 mg VSS/L SRB biomass and were kept shaken at 120 rpm, 37 °C. Control experiment was considered as the coupons incubated in a media containing 250 mg VSS/L SRB in the absence of CS/LS. The optimum concentration of CS/LS was directly added to the media in the range of 0–1000 µg·mL^−1^ CS/LS and EIS analysis was performed after 10 days of incubation. The *R_ct_* values from the Nyquist plots were used to evaluate the corrosion inhibition. Next, corrosion inhibition of optimum CS/LS concentration was evaluated at different time intervals (0, 7, 10, 15, 21, 28, and 35 days) [39,49,50]. The abiotic conditions were made by incubating coupons in the SRB-free media at sterile conditions and in absence of CS/LS to differentiate the chemical corrosion [51]. For comparison, 5% glutaraldehyde (GA) was used as a conventional biocide [52,53]. All experiments were performed in an anaerobic chamber at anaerobic conditions. After this, the sealed reaction bottles were kept shaking at different times at 37 °C. After particular time intervals, coupons were drawn from the incubation mixture and gently washed with DI water before analysis.

### 2.4. Electrochemical Measurements

Electrochemical measurements were recorded by Gamry potentiostat (Ref 600^+^, (Gamry Instruments, Warminster, PA, USA). Treated SS400 carbon-steel coupons were the working electrodes, saturated calomel electrode (SCE) as the reference and graphite disk as the counter electrode. The treated coupons were mounted into a Gamry ParaCell™ (Gamry Instruments, Warminster, PA, USA) Electrochemical Cell Kit (Part No.992-80) for electrochemical analysis. Simulated inject seawater was used as the electrolyte for all electrochemical experiments. The EIS measurements were recorded over 0.01–10^5^ Hz with 10 mV sinusoidal signal. The EIS measurements were performed after achieving the steady-state condition by keeping the setup for 30 min at open circuit potential (OCP). Gamry Echem Analyst software (Version 7.05) (Gamry Instruments, Warminster, PA, USA) was used to analyze the experimental data.

### 2.5. Biofilm, Corrosion Products, and Coupon Surface Characterization

For SEM and XPS analysis of SRB biofilm, the incubated coupons were fixed with 2% GA solution for 2 h. After washing the coupons with DI water, dehydration was performed with ethanol, followed by washing with water [39]. The coupons were stored under dry nitrogen before each analysis. The SEM and EDS analyses were performed with FEI Quanta 650 FEG (Hillsboro, OR, USA) SEM after gold (3 nm) coating. XPS analysis was carried out with ESCALAB 250X (Thermo Fisher Scientific, Hillsboro, OR, USA) with AlKα excitation (25 W, hυ = 1486.5 eV) and 1 eV resolution. The X-ray fluorescence (XRF) analysis was carried out with an XGT-7200V X-ray Analytical Microscope (Horiba Scientific, Piscataway, NJ, USA). The X-ray source was operated at 50 kV and 0.8 mA, and generates an X-ray beam from Rh anode that is focused to a spot size of 1.2 mm. To study the post corrosion morphology of carbon steel coupons, biofilms were removed by sonication in ethanol (10 s intervals), 5 mL·L^−1^ HCl, and 3.5 g·L^−1^ hexamethylenetetramine for 5 min followed by drying with nitrogen flow [54]. The post-corrosion morphology of the coupons after 35 days of incubation with and without CS/LS nanospheres were analyzed by SEM and profilometry. In addition, the bare coupon was analyzed for comparison. KLA-Tencor P17 stylus profilometer (Milpitas, CA, USA) at 2 µm resolution, with a loading force of 2 mg was used to capture surface profile images of the coupons. Seven measurements were made at each position on each coupon with a scan size of 400 × 400 µm^2^ for each frame. Apex 3d-7 software (Milpitas, CA, USA) was used to calculate the average surface roughness (*S_a_*).

## 3. Results and Discussion

### 3.1. Characterization of Cross-Linked CS/LS Nanospheres

**Figure 1:**
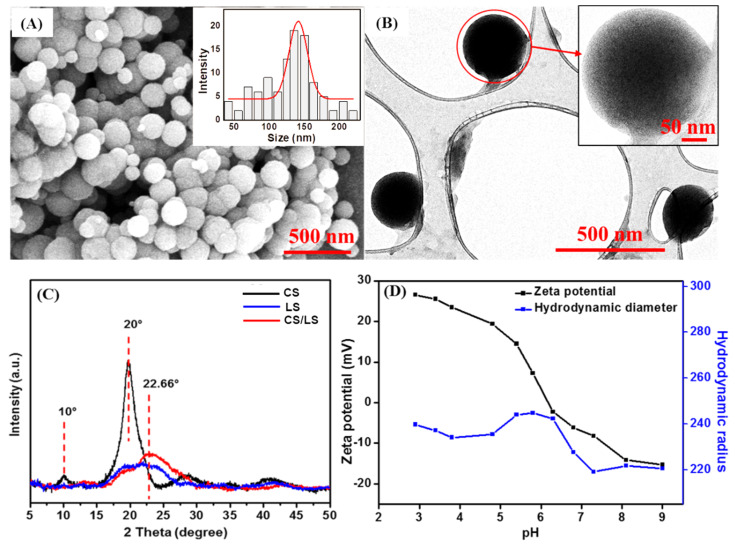
(**A**) SEM images of CS/LS nanospheres (Inset shows the size distribution); (**B**) TEM of CS/LS nanospheres (Inset: TEM image of a single nanosphere); (**C**) XRD for CS, LS, CS/LS and (**D**) The size and zeta potential changes with pH of the CS/LS in simulated seawater.

The uniform CS/LS nanospheres have been prepared by one-step covalent cross-linking between chitosan (CS) and lignosulfonate (LS) according to our reported method [31]. As shown in the SEM image (Figure 1A), well-dispersed spherical nanoparticles were obtained with an average diameter of 150–200 nm. TEM image further confirmed the well-defined shape of a single CS/LS nanosphere (Figure 1B). CS/LS at 1:1 CS:LS ratio has been considered as optimum ratio for preparing the nanospheres. According to the XRD pattern in Figure 1C, pure CS has two characteristic peaks at 10° and 20° while LS having a broad peak at 22.6° [55,56,57]. The formation of CS/LS is confirmed by the diminishing of the peak at 10°, as well as a reduction in intensity, and broadening of the peak at ~20°. This weaker and broader peak can be attributed to the formation of a new binary framework in which the original structure of both CS and LS can be disrupted. The detailed characterization of this material has been described elsewhere [31]. The average size distribution of CS/LS in the aqueous suspension was stable between 20 and 60 °C at pH 3–8 [31]. The stability of CS/LS in simulated seawater was evaluated by measuring the hydrodynamic radius and Zeta potential at varying pH between 3 and 9. The average size of the particles remained within the range of 230–250 nm at acidic media until pH 6. After this pH, the nanospheres have shrunk slightly and remained almost unchanged at pH above 7. This can be attributed to the collapse and deformation of CS/LS at alkaline pH values [31]. As shown in Figure 1D, the Zeta potential changed to a negative value with point of zero charge at pH 5.8, indicating that the CS/LS nanospheres are negatively charged at neutral pH conditions.

### 3.2. Investigation of SRB-Induced Corrosion on Carbon Steel

It is important to investigate the SRB induced MIC in our experimental conditions to make a valid comparison. The SS400 carbon steel coupons were incubated in a solution containing SRB in Postage’s C containing simulated seawater. SRB have been enriched from a mixed-culture bacterial sludge obtained from in a real oil filed sample (see experimental section). The coupons were analyzed by EIS after 7, 10, 15, 21, 28, and 35 days of incubation times.

**Figure 2:**
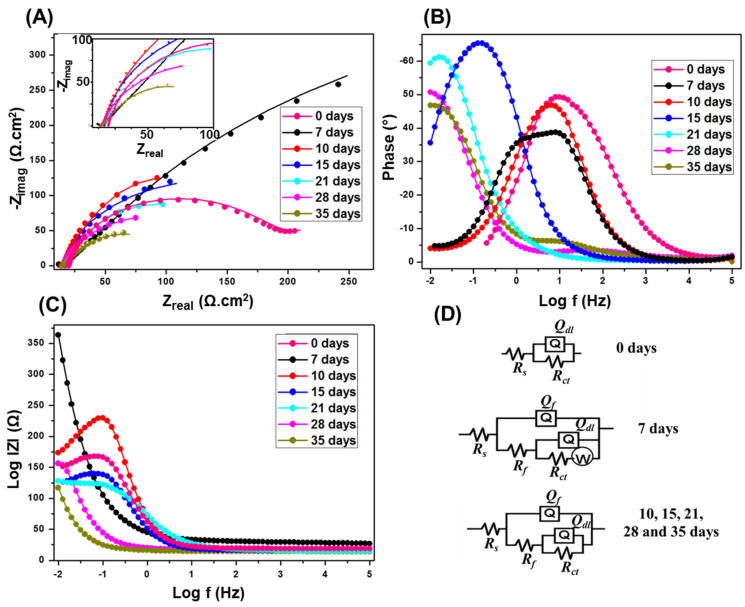
Nyquist (**A**); Bode (**B**,**C**) plots at SRB enriched media. The inset of (**A**) is a magnification of the low impedance region. The EIS were obtained at OCP of 10 mV and sinusoidal signal of 0.01–10^5^ Hz. (**D**) Equivalent circuits used to fit the experimental results.

As observed in Figure 2A, the *R_ct_* values of Nyquist semicircle is higher after 7 days compared to the ones from longer incubation times. A complete biofilm formation is expected to reach the highest protection capacity at 7 days [50,58]. After which, the semicircles diameters gradually decrease with time, indicating a gradual breakdown in the corrosion protection by the biofilm, i.e., faster corrosion rates. A small capacitive semicircle loop appeared at high frequencies, only for 7 days of incubation, mainly due to the precipitation of the corrosion product along with biofilm results in a porous and more adherent outer layer [59]. This high frequency semicircle started to diminish at longer incubation times, and the Nyquist plot behavior is also different compared to the longer incubation times. However, when steady state is reached at around 7 days, mass transfer limitations was dominant over the interfacial activation, which changes the shape of Nyquist from semicircle to a straight line in the low frequency regime.

The phase angle, θ, vs. frequency, in logarithmic scale plot (Figure 2B) presents the phase angle peak shifts to lower frequency with the increasing incubation times. The frequency shift confirms the increase in mass or thickness of the corrosion product layer with high electrical capacitance as a result of SRB activity onto carbon steel coupon [58]. Meanwhile, an increase in the capacitance is a result of increases in the mass and thickness of the porous layer, and consequently the surface area. From the impedance modulus |*Z*| vs. frequency, in logarithmic scale plot (Figure 2C), the |*Z*| values at low frequencies are maximum after 7 days, after which it decreases with longer incubation. The higher values of |*Z*| at low frequencies indicating lower corrosion rates [60].

Figure 2D represents the equivalent circuits of the fitted EIS data. *R_s_* represents the resistance of electrolyte, *Q_f_* is the constant phase element (CPE) at the film/solution interface, *R_f_* exemplify the pore film resistance, *Q_dl_* is the CPE of coupon/solution interface, *R_ct_* is the charge transfer resistance at the coupon/solution interface, and *W* represent Warburg impedance elements. Both circuits have constant phase elements (*Q_f_*) instead of the ideal electrical double layer capacitors. This was attributed to the surface roughness, inhomogeneous reaction rates distribution, non-uniform thickness, non-uniform composition of the double layer, and/or non-uniform current distribution [61,62,63,64,65]. Since the SRB biofilm provides a more prominent effect during the initial days of incubation, an additional element W is used in the equivalent circuit, corresponding to the diffusion controlled electrochemical process as a result of the complete biofilm formation [59]. As the incubation time increases, the biofilm starts to degrade so the mass transfer is not the controlling factor anymore, so W is removed from the equivalent circuit.

The EIS analysis of the coupons in the abiotic media in the absence CS/LS is shown in Figure S1. The semicircle’s behavior is different at 7 and 10 days in the absence of SRB compared to the longer incubation intervals. The precipitation of iron phosphide on the carbon steel surface can be detected from the change of medium frequency capacitive loops at 15 and 21 days of incubations [66]. Iron phosphide can be homogeneously distributed after precipitating with the ferrous ion produced by the steel dissolution under abiotic conditions [66]. A gradual decrease of the Nyquist plot diameter and the phase peak shift to a lower frequency was observed at a longer incubation time, and confirmed the low corrosion rate in the absence of SRB.

Table 1 gives the *R_ct_*, as well as *R_f_* values of the carbon steel coupons incubated in the presence of SRB after EIS fitting, and Supplementary Table S2 shows the complete EIS fitting data. The *R_ct_* value is highest at 7 days compared to other incubation times due to maximum protection of the complete biofilm. Afterward, the *R_ct_* values keep decreasing as the incubation time increases. The decrease in *R_ct_* value results in an increase in the dissolution kinetics of the metallic surface due to the fast corrosion rates induced by the breakdown of the biofilm that accelerates the corrosion process. Similarly, the *R_f_* values are highest at 7 days. The decrease in *R_f_* value could be a result of higher porosity of the biofilm on the coupon surface, resulting in the observed accelerated corrosion.

### 3.3. Investigation of CS/LS Nanospheres Inhibitory Effect on SRB Induced Corrosion

The previous aqueous media analysis indicated that CS/LS-1:1 demonstrates strong inhibition of SRB activities at 100 µg·mL^−1^. The CS/LS-1:1 demonstrated 48.8% inhibition of sulfate reduction and 54.26% reduction of total organic carbon (TOC) removal [31]. Here, we investigate the ability of the new CS/LS nanospheres to inhibit the biofilm formation and control MIC on the coupon surface. The first step was to identify the optimum concentration of CS/LS that gives maximum corrosion inhibition in a concentration range from 100 to 1000 µg·mL^−1^. SRB induced corrosion starts progressing after 10 days of incubation. Hence, the impedance analysis was performed after 10 days of incubation [39]. The Nyquist plots are shown in Supplementary Figure S2A and the equivalent circuit used for fitting the impedance plots is shown in Figure 2D. The relation between *R_ct_* values and CS/LS concentration are given in Supplementary Figure S2B. The *R_ct_* values of CS/LS are 256, 287, 337, 468 and 307 Ω·cm^2^ for 0, 100, 200, 500 and 1000 µg·mL^−1^ respectively. From the *R_ct_* values, it is found that 500 µg·mL^−1^ resulted in the maximum corrosion inhibition. At 1000 µg·mL^−1^, precipitation started to be observed in the reaction medium. Hence, 500 µg·mL^−1^ has been selected as the optimum concentrations of CS/LS for further experiments.

**Figure 3:**
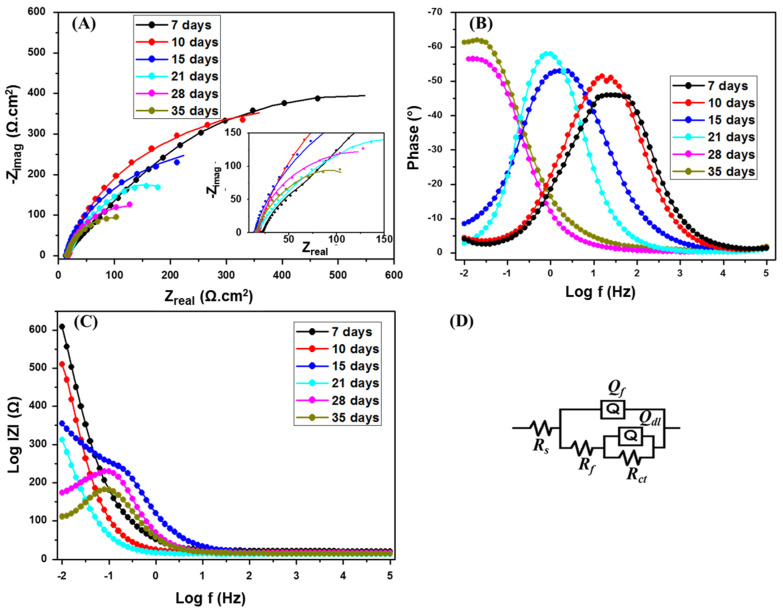
Nyquist (**A**); Bode (**B**,**C**) plots of SRB with CS/LS. The inset is a zoom of the low impedance region. The EIS were recorded at a range of 0.01–10^5^ Hz. (**D**) Equivalent circuit used for fitting.

The effect of incubation time on the carbon steel coupons was investigated by EIS after 7, 10, 15, 21, 28, and 35 days in the media containing SRB at 500 µg·mL^−1^ CS/LS. The Nyquist plot (Figure 3A) showed the same trend as in the presence of SRB but the diameter is higher at the corresponding incubation times. The equivalent circuit used is shown in Figure 3D. The increase in the diameter of the semicircle in the impedance spectrum implies corrosion inhibition in the presence of CS/LS. The *R_ct_* values are 609 and 363.6 Ω·cm^2^ respectively (Table 1) for the coupon incubated with and without CS/LS respectively after 7 days. However, the Nyquist plot behavior is different for 7 days of incubation compared to the longer incubation periods, and there is no high-frequency capacitive loop after 7 days or even at higher incubation times in presence of CS/LS [59,67]. This can be attributed to the formation of CS/LS layer on the metal surface as also confirmed by the increase in *R_f_* values.

From the phase angle θ vs. frequency, in the logarithmic scale plot (Figure 3B), the phase peak has shifted to a lower frequency with increasing incubation time similar to the SRB corrosion experiments. However, the intensity of the lower frequency shift is less compared to the presence of SRB. Similarly, from the impedance modulus |*Z*| vs. frequency, in logarithmic scale plot (Figure 3C), the |*Z*| value at low frequencies is high in the case of 7 days incubation.

The *R_ct_* value is maximum at 7 days and it has decreasing with the higher incubation time (Table 1). However, the *R_ct_* values are about two times more than the SRB corrosion rate at the corresponding incubation intervals and this enhancement in the *R_ct_* value is due to the corrosion inhibition effect of CS/LS. The inhibitory effect of CS/LS in absence of the SRB is evaluated after 7 days of incubation (Supplementary Figure S3). The CS/LS may compete with the biofilm and form a spatial layer on the coupon surface, and this can be verified by comparing the R*_f_* values during the initial incubation times [67]. The *R_f_* values are 3–3.7 times higher in the presence of CS/LS compared with SRB alone during 7–15 days of incubation. The biofilm breakdown takes place as time lapses, which is confirmed by the decrease in the *R_f_* values with longer incubation times.

Corrosion inhibition efficiency (IE) is obtained from:

IE = (*R_ct’_* − *R_ct_*)/*R_ct’_* (1)

where *R_ct’_* is obtained in the presence of SRB with CS/LS and *R_ct_* is in the presence of SRB alone. The IE at different incubation time intervals is given in Table 1. The maximum corrosion inhibition was found to be 85% in presence of CS/LS. In our previous study, the CZNC inhibitor was able to provide 74% maximum corrosion inhibition with lesser dose of 250 µg·mL^−1^ [24]. There was no significant enhancement in the corrosion inhibition, even at the higher dose of 500 µg·mL^−1^. However, the inhibitor dose of CZNC was limited to 250 µg·mL^−1^ due to the ZnO content in the CZNC biocide. Here we were able to use a higher dose of 500 µg·mL^−1^ since CS/LS are metal-free and made of renewable components with expected low toxicity. Nevertheless, the toxicity range and environmental impact of the new nanospheres need to be investigated in future studies.

The corrosion inhibition capability of CS/LS has been compared with the commercial GA biocide by EIS analysis. Supplementary Figure S4 shows the Nyquist plots of the coupons incubated in 5% GA after 15 days of incubation showing a lower diameter of the semicircle in the impedance spectrum as compared with CS/LS, which can be attributed to the better corrosion inhibition induced by the CS/LS. The *R_f_* and *R_ct_* values are low (136.4 and 158.8 Ω·cm^2^, respectively) when SRB is incubated with GA as compared with CS/LS (209 and 312.8 Ω·cm^2^, respectively). In the case of SRB with 5% GA, complete bacterial growth can be inhibited; however, a side reaction can be observed between SRB media and GA which is evidenced by the change of media color to pink instead of the expected black (Supplementary Figure S4). Despite the highly efficiency of GA as a biocide, its toxicity effect to the aquatic ecosystems limited their use [6]. Moreover, GA did not entirely suppress the corrosion in the studied medium during longer incubation times, most likely due to the accumulation of some corrosion products in the cracks of the carbon steel surface. Therefore, CS/LS can provide a more benign alternative to minimize or replace the utilization of GA as a biocide.

### 3.4. Biofilm and Corrosion Products Characterization

**Figure 4:**
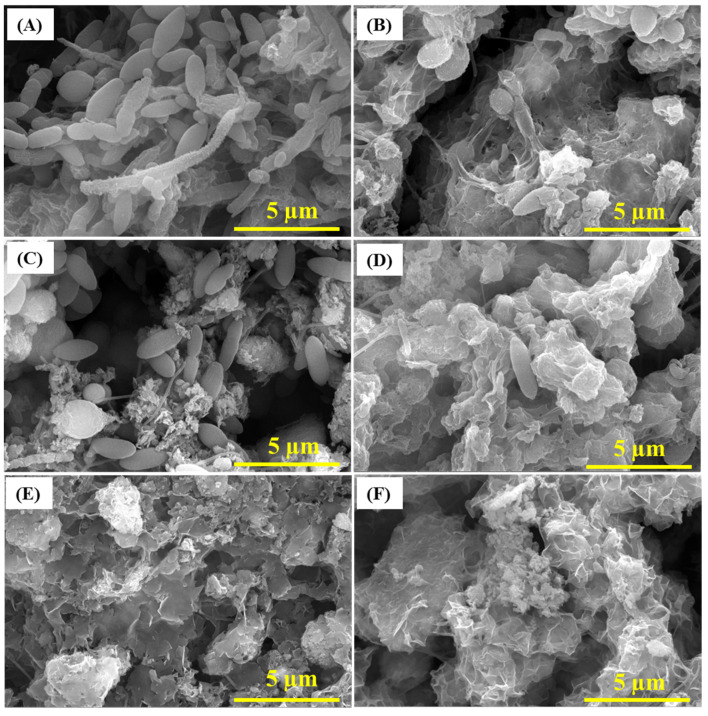
SEM images of the biofilm after 7 days in SRB media (**A**), 21 days (**C**) and 35 days (**E**) of incubations. SEM of the biofilm after 7 (**B**), 21 (**D**) and 35 days (**F**) in SRB media containing 500 µg·mL^−1^ CS/LS.

The inhibition of SRB by CS/LS (500 µg·mL^−1^) and the consequent formation of biofilm and corrosion products on the carbon steel were examined by SEM, EDS, and XPS analysis. Generally, the presence of exopolysaccharides (EPS), which are excreted by the bacteria to adhere to the metal surface, is visible after four days of incubation along with SRB cells (Supplementary Figure S5). In the presence of CS/LS, different morphology of EPS is visible by SEM due to the possible complex formation with the exopolysaccharide component of CS/LS. Few SRB cells are present on the surface but with deformed cell morphology (Figure S5). After 7 days, the adhesion of numerous active SRBs can be seen on the coupon surface (Figure 4A). Meanwhile, the number of attached SRB cells is significantly reduced in presence of CS/LS, even with noticeable damage at the bacterium cell surface (Figure 4B). The CS/LS particles on the metal surface may have hindered the bacterial attachment as indicated by the smaller number of bacteria on the surface. In addition, an obvious damage to the bacterial cell walls can be seen [31]. Similar observations of cell wall damage were observed as the effect of different nanomaterials on other bacteria [28,68]. For example, predominant damage can be induced on most of the SRB cells in presence of 100 µg·mL^−1^ of ZnONPs [28]. Another work reported a progressive damage to the cell wall of *Staphylococcus aureus* and *Pseudomonas aeruginosa*, causing a total lysis of cells in contact with the nanocomposites [68]. Chitosan-based CZNC-10 showed a similar behavior on biofilm formation which was attributed to the synergic influence of CZNC-10 bactericidal impact on the SRB together with the formation of a protective coating on the coupon surface [6,24,67].

After 21 days, uneven deposits of corrosion products were visible on the coupon surface along with complex porous structure of the biofilm when exposed to SRB alone (Figure 4C) [69]. In the presence of CS/LS, SEM showed different corrosion products and biofilm morphology with few deformed bacteria on the surface (Figure 4D). After 35 days, corrosion products were dominant on the surface along with limited biofilm structures in the case of SRB alone (Figure 4E,F). In the presence of CS/LS, similar observation is present but with different morphology of the corrosion products. No bacteria were visible on the coupon surface in both cases, which could be covered by the corrosion products layer. The different morphology is an implication of heterogeneous corrosion products in presence of the CS/LS nanoparticles. EDS and XRF analysis have quantified the sulfur and iron content in both biofilm and corrosion products. According to the EDS analysis after 35 days, a reduction in the concentration of Fe and S content by 43% and 31% respectively was observed in the presence of CS/LS (Figure S6). The XRF analysis also showed the reduction in Fe and S contents by 56% and 29%, respectively, in the presence of CS/LS, confirming the inhibition of SRB (Supplementary Table S3).

**Figure 5:**
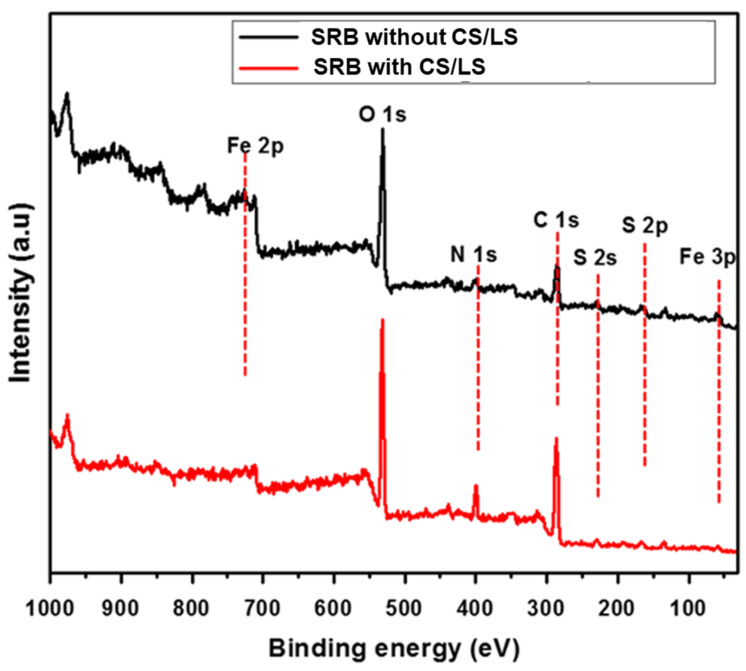
XPS survey spectra of the coupon surface incubated in SRB enriched media with and without CS/LS after 35 days incubation.

Figure 5 shows the XPS survey of corrosion products after 35 days in SRB media with and without 500 µg·mL^−1^ of CS/LS. SRB induced biocorrosion follows the sulfate reduction pathway at the metal surface with the help of a hydrogen intermediate [70]. This sulfide can appear as H_2_S, HS^−^ ions, S^2−^ ions or metal sulfides, according to the different conditions which build up at the metal surface and catalyze the corrosion process [71]. As a result, the Fe is oxidized to Fe^2+^ and sulfate is reduced to sulfide followed by the formation of FeS. The overall reaction can be written as:

4 Fe^0^ + SO_4_^2−^ + 3 HCO_3_^−^ + 5 H^+^ → FeS + 3 FeCO_3_ + 4 H_2_O

The peaks of Fe 3p, Fe 2p, C 1s, O 1s, S 2p, and S 2s are observed in both spectra, which can be attributed to corrosion products and biofilm on the coupon. The S 2p and S 2s peaks confirmed the presence of sulfide and organic sulfur which are mainly formed by the SRB activity. However, the smaller peaks of Fe 2p, S 2s and S 2p were observed from coupon incubated with CS/LS. In addition, the high-resolution spectra for Fe 2p and S 2p are examined to confirm the reduction in the intensity of XPS peak as well as to quantify the corrosion products.

**Figure 6:**
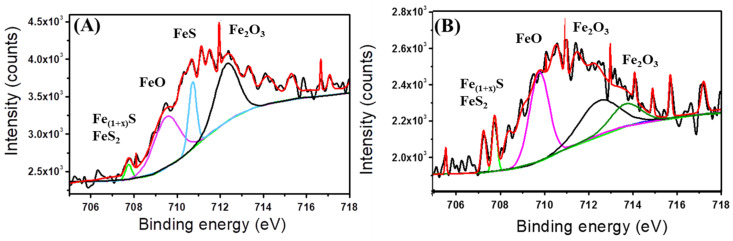
Fitted Fe 2p spectra from coupons incubated in SRB enriched media without CS/LS (**A**) and with 500 µg·mL^−1^ CS/LS (**B**) after 35 days of incubation. Fitted curves with different colour correspond to different chemical states; light green corresponds to FeS_2_ and mackinawite (Fe_1_ + _x_S), pink corresponds to FeO, Cyan corresponds to FeS, black and green correspond to Fe_2_O_3_.

The fitted Fe 2p peaks after 35 days with and without CS/LS are shown in Figure 6A,B respectively. Two sharp peaks at 709.6 eV and 707.6 eV (Fe 2p_3/2_) corresponding to FeO (pink curve) and mackinawite (Fe_1+x_S) (green curve), respectively. Pyrite (FeS_2_) is present in both spectra (green curve) [50]. In addition, a peek at around 712.4 eV corresponds to Fe^3+^ (originated from Fe_2_O_3_) is present in both the coupons (black curve) [72]. A sharp peak at around 710.4 eV (Fe 2p_3/2_) corresponding to FeS (cyan curve) is present in the coupon exposed to SRB alone [73]. The peak at 713.7 eV of (Fe 2p_3/2_) corresponds to Fe(III)O from Fe_2_O_3_ and is found in presence of CS/LS [74]. From the XPS analysis, the corrosion products are mostly FeO, FeS, FeS_2_, and Fe_2_O_3_. However, FeS peak is prominent only in the coupon exposed to SRB alone which confirmed the reduction in SRB activity in the presence of CS/LS.

**Figure 7:**
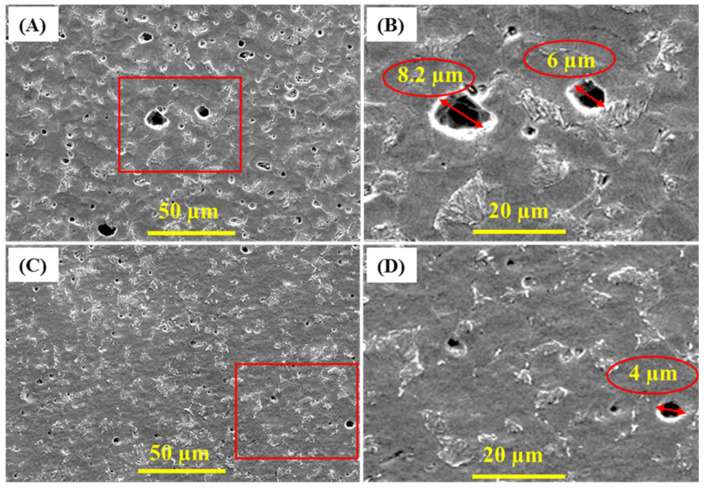
SEM micrograph of corrosion pits after removing the corrosion products from SS400 coupons incubated in SRB alone (**A**,**B**) and in presence of CS/LS nanospheres (**C**,**D**) after 35 days of incubation.

SEM and surface profilometry were used to analyze the coupon surface after removing biofilms and corrosion products [75,76,77]. Figure 7 shows the formed pits on the coupon surface. Pits diameter is greater when coupon is incubated in SRB alone as compared with SRB/CS/LS mixture. The widest pit is observed in the coupon incubated with SRB alone is around 8.2 µm diameter while it is only 4 µm in the presence of CS/LS. These results are matching with EIS and XPS data. In addition, the surface roughness of both coupons was also calculated form profilometry. For comparison, the 2D and 3D profilometry images of the bare coupon is shown in Supplementary Figure S7A,B. The average roughness of the bare coupon is 17 ± 2 nm, which is appropriate for bacterial attachment [78,79,80]. Supplementary Figure S8 displays high-resolution spectra of the coupon surface after removing the corrosion products. After 35 days, the average roughness of the coupons surface was 780 ± 19 nm in the absence of CS/LS inhibitor. While in the presence of CS/LS inhibitor, the average surface roughness is reduced to 458 ± 16 nm, i.e., the surface roughness of the coupon is reduced to approx. 40% in presence of the CS/LS.

In general, the corrosion inhibition mechanism of nanomaterials can be originated mainly from their antibacterial effect [6]. The CS/LS antibacterial activity can be explained by their surface charge and active surface of CS and LS [31]. Due to the nanostructure and hydrophilic nature of CS/LS (originated from the presence of large number of amino groups of CS component), CS/LS can readily bind to the negatively charged bacteria, leading to cell membrane disruption [24]. Lignosulfonate component can cause oxidative stress to proteins and DNA in bacteria generated by reactive oxygen species. These two processes can cause a reduction in the cell viability by severe cytoplasmic leakage and loss of cell integrity and EPS contents. Sulfate reduction and co-substrate oxidation assays confirmed the CS/LS inhibitory effect on SRB [31]. The SEM analysis of SRB cells showed cell aggregation and prevalent surface damage after CS/LS exposure [31]. In addition, formation of CS/LS layer on the coupon surface protects the surface from the initial bacterial attachment [81]. Morphological results and EIS experiments suggest that CS/LS have hindered the formation of biofilm and unstable corrosion products on the carbon steel surface.

## 4. Conclusions

CS/LS nanospheres have been successfully evaluated as a novel biocide for the inhibition of SRB induced biocorrosion. The *R_ct_* values are approximately doubled in the presence of CS/LS compared with the CS/LS-free media, irrespective of incubation intervals from the electrochemical analysis, with a corrosion inhibition efficiency of 85% at 500 µg·mL^−1^ CS/LS. Post-corrosion analysis with SEM and profilometry showed fewer surface defects on the coupon incubated with CS/LS, indicating less corrosion. Two synergic effects can explain the biocidal effect of CS/LS. The hydrophilic CS/LS can readily bind to the bacterial surfaces and thereby damage the bacterial cell wall. Meanwhile, the film forming capability of CS prevents the initial bacterial attachment on the metal surface and thereby leads to a reduction of biofilm formation. In short, due to the biodegradable nature, promising antimicrobial properties of the building blocks, and high biocorrosion inhibition efficiency, the CS/LS nanospheres can present a renewable, cost efficient, and environmentally benign biocide for the inhibition of SRB induced MIC on carbon steel systems.

## Figures and Tables

**Table 1:** *R_ct_*, R_f_ and IE values after EIS fitting.

Incubation Media	Incubation Time (Days)	*R_f_* (Ω·cm^2^)	*R_ct_* (Ω·cm^2^)	IE (%)
SRB alone	7	79.7	363.6	-
	10	75.6	256.1	-
	15	69.4	173.8	-
	21	45.4	137.2	-
	28	38.6	107.9	-
	35	23.6	88.4	-
CS/LS alone	7	136.1	74.4	
SRB with CS/LS	7	292.7	609	68
	10	249.2	468	82
	15	209	312.8	80
	21	129.7	254.6	85
	28	90.6	193.2	80
	35	71.2	157.3	78

## Supplementary Materials

The following are available online at https://www.mdpi.com/1996-1944/13/11/2484/s1, Figure S1: The Nyquist plot of the coupon incubated in the abiotic media (A). The corresponding Bode plots are given in (B) and (C). Figure S2: (A) The Nyquist plot of the coupon incubated with SRB with CS/LS with different concentrations from 0 to 1000 µg·mL^−1^. The impedance analysis was performed after 10 days of incubation. (B) The *R_ct_* vs concentration of CS/LS after 10 days of incubation. The error bar indicates the standard deviation from the three independent measurements. Figure S3: The Nyquist plot of the coupon after 7 days of incubation in presence of CS/LS. Figure S4: Nyquist plot of the incubated coupon after 15 days in SRB enriched media with 5% GA. Inset shows the fitting equivalent circuit and the photographs of the incubation mixtures after 15 days of incubation. Figure S5: SEM images of the biofilm grown on the coupon surface after being incubated with SRB enriched media for 4 days without inhibitor (A) and in presence of 500 µg·mL^−1^ CS/LS (B). Figure S6: EDS analysis of biofilm incubated in SRB enriched media without CS/LS (A) and with 500 µg·mL^−1^ CS/LS (B) after 35 days of incubation. Figure S7: Profilometry of the bare carbon steel coupon surface. (A) 2D and (B) 3D images. Figure S8: Profilometry images (2D and 3D) of the cleaned carbon steel coupon surface after 35 days of incubation in SRB without CS/LS (A and B) and SRB with 500 µg·mL^−1^ CS/LS (C and D), respectively. Table S1: Composition of simulated seawater. Table S2. EIS fitting data. Table S3: XRF data of the biofilm incubated in SRB enriched media without CS/LS (A) and with 500 µg·mL^−1^ CS/LS (B) after 35 days of incubation.

## References

[1] Vanaei H.R., Eslami A., Egbewande A. (2017). A review on pipeline corrosion, in-line inspection (ILI), and corrosion growth rate models. Int. J. Press. Vessel. Pip..

[2] Xu D., Huang W., Ruschau G., Hornemann J., Wen J., Gu T. (2013). Laboratory investigation of MIC threat due to hydrotest using untreated seawater and subsequent exposure to pipeline fluids with and without SRB spiking. Eng. Fail. Anal..

[3] Skovhus T.L., Enning D., Lee J.S. (2017). Microbiologically Influenced Corrosion in the Upstream Oil and Gas Industry.

[4] Wang H., Wang Z., Hong H., Yin Y. (2010). Preparation of cerium-doped TiO2 film on 304 stainless steel and its bactericidal effect in the presence of sulfate-reducing bacteria (SRB). Mater. Chem. Phys..

[5] Enning D., Venzlaff H., Garrelfs J., Dinh H.T., Meyer V., Mayrhofer K., Hassel A.W., Stratmann M., Widdel F. (2012). Marine sulfate-reducing bacteria cause serious corrosion of iron under electroconductive biogenic mineral crust. Environ. Microbiol..

[6] Rasheed P.A., Jabbar K.A., Mackey H.R., Mahmoud K.A. (2019). Recent advancements of nanomaterials as coatings and biocides for the inhibition of sulfate reducing bacteria induced corrosion. Curr. Opin. Chem. Eng..

[7] Rajasekar A., Anandkumar B., Maruthamuthu S., Ting Y.P., Rahman P. (2010). Characterization of corrosive bacterial consortia isolated from petroleum-product-transporting pipelines. Appl. Microbiol. Biotechnol..

[8] Vance I., Thrasher D.R. (2005). Reservoir souring: Mechanisms and prevention.

[9] Enning D., Garrelfs J. (2014). Corrosion of Iron by Sulfate-Reducing Bacteria: New Views of an Old Problem. Appl. Environ. Microbiol..

[10] Sun C., Xu J., Wang F. (2011). Interaction of Sulfate-Reducing Bacteria and Carbon Steel Q 235 in Biofilm. Ind. Eng. Chem. Res..

[11] Antony P.J., Raman R.K.S., Raman R., Kumar P. (2010). Role of microstructure on corrosion of duplex stainless steel in presence of bacterial activity. Corros. Sci..

[12] Esquivel R.G., Olivares G.Z., Gayosso M.J.H., Trejo A.G. (2011). Cathodic protection of XL 52 steel under the influence of sulfate reducing bacteria. Mater. Corros..

[13] Krishnamurthy A., Gadhamshetty V., Mukherjee R., Chen Z., Ren W., Cheng H.M., Koratkar N. (2013). Passivation of microbial corrosion using a graphene coating. Carbon.

[14] Duncan K.E., Perez-Ibarra B.M., Jenneman G., Harris J.B., Webb R., Sublette K. (2014). The effect of corrosion inhibitors on microbial communities associated with corrosion in a model flow cell system. Appl. Microbiol. Biotechnol..

[15] Narenkumar J., Parthipan P., Usha Raja Nanthini A., Benelli G., Murugan K., Rajasekar A. (2017). Ginger extract as green biocide to control microbial corrosion of mild steel. 3 Biotech..

[16] Xue Y., Voordouw G. (2015). Control of Microbial Sulfide Production with Biocides and Nitrate in Oil Reservoir Simulating Bioreactors. Front. Microbiol..

[17] Nguyen T., Roddick F.A., Fan L. (2012). Biofouling of water treatment membranes: A review of the underlying causes, monitoring techniques and control measures. Membranes.

[18] Rasool K., Helal M., Ali A., Ren C.E., Gogotsi Y., Mahmoud K.A. (2016). Antibacterial Activity of Ti3C2Tx MXene. ACS Nano.

[19] Hajipour M.J., Fromm K.M., Akbar Ashkarran A., Jimenez de Aberasturi D., Larramendi I.R.d., Rojo T., Serpooshan V., Parak W.J., Mahmoudi M. (2012). Antibacterial properties of nanoparticles. Trends Biotechnol..

[20] Mathur A., Bhuvaneshwari M., Babu S., Chandrasekaran N., Mukherjee A. (2017). The effect of TiO2 nanoparticles on sulfate-reducing bacteria and their consortium under anaerobic conditions. J. Environ. Chem. Eng..

[21] Khowdiary M.M., El-Henawy A.A., Shawky A.M., Sameeh M.Y., Negm N.A. (2017). Synthesis, characterization and biocidal efficiency of quaternary ammonium polymers silver nanohybrids against sulfate reducing bacteria. J. Mol. Liq..

[22] Fathy M., Badawi A., Mazrouaa A.M., Mansour N.A., Ghazy E.A., Elsabee M.Z. (2013). Styrene N-vinylpyrrolidone metal-nanocomposites as antibacterial coatings against Sulfate Reducing Bacteria. Mater. Sci. Eng. C.

[23] Wan D., Yuan S., Neoh K.G., Kang E.T. (2010). Surface Functionalization of Copper via Oxidative Graft Polymerization of 2,2′-Bithiophene and Immobilization of Silver Nanoparticles for Combating Biocorrosion. Acs Appl. Mater. Interfaces.

[24] Rasool K., Nasrallah G.K., Younes N., Pandey R.P., Abdul Rasheed P., Mahmoud K.A. (2018). “Green” ZnO-Interlinked Chitosan Nanoparticles for the Efficient Inhibition of Sulfate-Reducing Bacteria in Inject Seawater. Acs Sustain. Chem. Eng..

[25] Kumar N., Omoregie E.O., Rose J., Masion A., Lloyd J.R., Diels L., Bastiaens L. (2014). Inhibition of sulfate reducing bacteria in aquifer sediment by iron nanoparticles. Water Res..

[26] Krishnamurthy A., Gadhamshetty V., Mukherjee R., Natarajan B., Eksik O., Ali Shojaee S., Lucca D.A., Ren W., Cheng H.-M., Koratkar N. (2015). Superiority of Graphene over Polymer Coatings for Prevention of Microbially Induced Corrosion. Sci. Rep..

[27] Alasvand Zarasvand K., Rai V.R. (2016). Inhibition of a sulfate reducing bacterium, Desulfovibrio marinisediminis GSR3, by biosynthesized copper oxide nanoparticles. 3 Biotech..

[28] Rasool K., Lee D.S. (2016). Effect of ZnO nanoparticles on biodegradation and biotransformation of co-substrate and sulphonated azo dye in anaerobic biological sulfate reduction processes. Int. Biodeterior. Biodegrad..

[29] Yan X., Rong R., Zhu S., Guo M., Gao S., Wang S., Xu X. (2015). Effects of ZnO Nanoparticles on Dimethoate-Induced Toxicity in Mice. J. Agric. Food Chem..

[30] Ashraf M.A., Ullah S., Ahmad I., Qureshi A.K., Balkhair K.S., Abdur Rehman M. (2014). Green biocides, a promising technology: Current and future applications to industry and industrial processes. J. Sci. Food Agric..

[31] Pandey R.P., Rasool K., Rasheed P.A., Gomez T., Pasha M., Mansour S.A., Lee O.-S., Mahmoud K.A. (2020). One-step synthesis of an antimicrobial framework based on covalently cross-linked chitosan/lignosulfonate (CS@LS) nanospheres. Green Chem..

[32] Hosseinnejad M., Jafari S.M. (2016). Evaluation of different factors affecting antimicrobial properties of chitosan. Int. J. Biol. Macromol..

[33] Yuan G., Lv H., Tang W., Zhang X., Sun H. (2016). Effect of chitosan coating combined with pomegranate peel extract on the quality of Pacific white shrimp during iced storage. Food Control..

[34] Piras A.M., Maisetta G., Sandreschi S., Gazzarri M., Bartoli C., Grassi L., Esin S., Chiellini F., Batoni G. (2015). Chitosan nanoparticles loaded with the antimicrobial peptide temporin B exert a long-term antibacterial activity in vitro against clinical isolates of Staphylococcus epidermidis. Front. Microbiol..

[35] Ramezani Z., Zarei M., Raminnejad N. (2015). Comparing the effectiveness of chitosan and nanochitosan coatings on the quality of refrigerated silver carp fillets. Food Control..

[36] Zhang A., Mu H., Zhang W., Cui G., Zhu J., Duan J. (2013). Chitosan Coupling Makes Microbial Biofilms Susceptible to Antibiotics. Sci. Rep..

[37] Martinez L.R., Mihu M.R., Han G., Frases S., Cordero R.J.B., Casadevall A., Friedman A.J., Friedman J.M., Nosanchuk J.D. (2010). The use of chitosan to damage Cryptococcus neoformans biofilms. Biomaterials.

[38] Andersen T., Mishchenko E., Flaten G., Sollid J., Mattsson S., Tho I., Škalko-Basnet N. (2017). Chitosan-Based Nanomedicine to Fight Genital Candida Infections: Chitosomes. Mar. Drugs.

[39] Rasheed P.A., Jabbar K.A., Rasool K., Pandey R.P., Sliem M.H., Helal M., Samara A., Abdullah A.M., Mahmoud K.A. (2019). Controlling the biocorrosion of sulfate-reducing bacteria (SRB) on carbon steel using ZnO/chitosan nanocomposite as an eco-friendly biocide. Corros. Sci..

[40] Azadi P., Inderwildi O.R., Farnood R., King D.A. (2013). Liquid fuels, hydrogen and chemicals from lignin: A critical review. Renew. Sustain. Energy Rev..

[41] Berlin A., Balakshin M., Gupta V.K., Tuohy M.G., Kubicek C.P., Saddler J., Xu F. (2014). Chapter 18-Industrial Lignins: Analysis, Properties, and Applications. Bioenergy Research: Advances and Applications.

[42] Kim S., Fernandes M.M., Matamá T., Loureiro A., Gomes A.C., Cavaco-Paulo A. (2013). Chitosan–lignosulfonates sono-chemically prepared nanoparticles: Characterisation and potential applications. Colloids Surf. B Biointerfaces.

[43] Lora J.H., Glasser W.G. (2002). Recent Industrial Applications of Lignin: A Sustainable Alternative to Nonrenewable Materials. J. Polym. Environ..

[44] Dong X., Dong M., Lu Y., Turley A., Jin T., Wu C. (2011). Antimicrobial and antioxidant activities of lignin from residue of corn stover to ethanol production. Ind. Crops Prod..

[45] Fredheim G.E., Christensen B.E. (2003). Polyelectrolyte Complexes:  Interactions between Lignosulfonate and Chitosan. Biomacromolecules.

[46] Al-Rashed M.M., Niknezhad S., Jana S.C. (2019). Mechanism and Factors Influencing Formation and Stability of Chitosan/Lignosulfonate Nanoparticles. Macromol. Chem. Phys..

[47] Yan M., Huang W., Li Z. (2019). Chitosan cross-linked graphene oxide/lignosulfonate composite aerogel for enhanced adsorption of methylene blue in water. Int. J. Biol. Macromol..

[48] Javed M.A., Neil W.C., McAdam G., Wade S.A. (2017). Effect of sulphate-reducing bacteria on the microbiologically influenced corrosion of ten different metals using constant test conditions. Int. Biodeterior. Biodegrad..

[49] Javed M.A., Stoddart P.R., Wade S.A. (2015). Corrosion of carbon steel by sulphate reducing bacteria: Initial attachment and the role of ferrous ions. Corros. Sci..

[50] Yuan S., Liang B., Zhao Y., Pehkonen S.O. (2013). Surface chemistry and corrosion behaviour of 304 stainless steel in simulated seawater containing inorganic sulphide and sulphate-reducing bacteria. Corros. Sci..

[51] Huttunen-Saarivirta E., Rajala P., Carpén L. (2016). Corrosion behaviour of copper under biotic and abiotic conditions in anoxic ground water: Electrochemical study. Electrochim. Acta.

[52] Yin B., Williams T., Koehler T., Morris B., Manna K. (2018). Targeted microbial control for hydrocarbon reservoir: Identify new biocide offerings for souring control using thermophile testing capabilities. Int. Biodeterior. Biodegrad..

[53] Kahrilas G.A., Blotevogel J., Stewart P.S., Borch T. (2015). Biocides in Hydraulic Fracturing Fluids: A Critical Review of Their Usage, Mobility, Degradation, and Toxicity. Environ. Sci. Technol..

[54] Chen S.Q., Wang P., Zhang D. (2016). The influence of sulphate-reducing bacteria on heterogeneous electrochemical corrosion behavior of Q235 carbon steel in seawater. Mater. Corros..

[55] Wang Y., Pitto-Barry A., Habtemariam A., Romero-Canelon I., Sadler P.J., Barry N.P.E. (2016). Nanoparticles of chitosan conjugated to organo-ruthenium complexes. Inorg. Chem. Front..

[56] Li P.-C., Liao G.M., Kumar S.R., Shih C.-M., Yang C.-C., Wang D.-M., Lue S.J. (2016). Fabrication and Characterization of Chitosan Nanoparticle-Incorporated Quaternized Poly(Vinyl Alcohol) Composite Membranes as Solid Electrolytes for Direct Methanol Alkaline Fuel Cells. Electrochim. Acta.

[57] Wang C.H., Fang G.Z., Ai Q., Zhao Y.F. (2011). Preparation of Lignosulfonate-Chitosan Polyelectrolyte Complex. Adv. Mater. Res..

[58] AlAbbas F.M., Williamson C., Bhola S.M., Spear J.R., Olson D.L., Mishra B., Kakpovbia A.E. (2013). Influence of sulfate reducing bacterial biofilm on corrosion behavior of low-alloy, high-strength steel (API-5L X80). Int. Biodeterior. Biodegrad..

[59] Castaneda H., Benetton X.D. (2008). SRB-biofilm influence in active corrosion sites formed at the steel-electrolyte interface when exposed to artificial seawater conditions. Corros. Sci..

[60] Su C., Wu W., Li Z., Guo Y. (2015). Prediction of film performance by electrochemical impedance spectroscopy. Corros. Sci..

[61] Kim C.-H., Pyun S.-I., Kim J.-H. (2003). An investigation of the capacitance dispersion on the fractal carbon electrode with edge and basal orientations. Electrochim. Acta.

[62] Mulder W.H., Sluyters J.H., Pajkossy T., Nyikos L. (1990). Tafel current at fractal electrodes: Connection with admittance spectra. J. Electroanal. Chem. Interfacial Electrochem..

[63] Schiller C.A., Strunz W. (2001). The evaluation of experimental dielectric data of barrier coatings by means of different models. Electrochim. Acta.

[64] Jorcin J.-B., Orazem M.E., Pébère N., Tribollet B. (2006). CPE analysis by local electrochemical impedance spectroscopy. Electrochim. Acta.

[65] Oldham K.B. (2004). The RC time “constant” at a disk electrode. Electrochem. Commun..

[66] Liu H., Fu C., Gu T., Zhang G., Lv Y., Wang H., Liu H. (2015). Corrosion behavior of carbon steel in the presence of sulfate reducing bacteria and iron oxidizing bacteria cultured in oilfield produced water. Corros. Sci..

[67] Gupta N.K., Joshi P.G., Srivastava V., Quraishi M.A. (2018). Chitosan: A macromolecule as green corrosion inhibitor for mild steel in sulfamic acid useful for sugar industry. Int. J. Biol. Macromol..

[68] Regiel-Futyra A., Kus-Liśkiewicz M., Sebastian V., Irusta S., Arruebo M., Stochel G., Kyzioł A. (2015). Development of Noncytotoxic Chitosan–Gold Nanocomposites as Efficient Antibacterial Materials. Acs Appl. Mater. Interfaces.

[69] Chen S., Wang P., Zhang D. (2014). Corrosion behavior of copper under biofilm of sulfate-reducing bacteria. Corros. Sci..

[70] Lin J., Ballim R. (2012). Biocorrosion control: Current strategies and promising alternatives. Afr. J. Biotechnol..

[71] Kan J., Chellamuthu P., Obraztsova A., Moore J.E., Nealson K.H. (2011). Diverse bacterial groups are associated with corrosive lesions at a Granite Mountain Record Vault (GMRV). J. Appl. Microbiol..

[72] Grosvenor A.P., Kobe B.A., Biesinger M.C., McIntyre N.S. (2004). Investigation of multiplet splitting of Fe 2p XPS spectra and bonding in iron compounds. Surf. Interface Anal..

[73] Zheng B., Li K., Liu H., Gu T. (2014). Effects of Magnetic Fields on Microbiologically Influenced Corrosion of 304 Stainless Steel. Ind. Eng. Chem. Res..

[74] Wang W.P., Yang H., Xian T., Jiang J.L. (2012). XPS and magnetic properties of CoFe_2_O_4_ nanoparticles synthesized by a polyacrylamide gel route. Mater. Trans..

[75] Finšgar M. (2013). 2-Mercaptobenzimidazole as a copper corrosion inhibitor: Part, I. Long-term immersion, 3D-profilometry, and electrochemistry. Corros. Sci..

[76] Finšgar M., Merl D.K. (2014). 2-Mercaptobenzoxazole as a copper corrosion inhibitor in chloride solution: Electrochemistry, 3D-profilometry, and XPS surface analysis. Corros. Sci..

[77] Xu D.K., Birbilis N., Lashansky D., Rometsch P.A., Muddle B.C. (2011). Effect of solution treatment on the corrosion behaviour of aluminium alloy AA7150: Optimisation for corrosion resistance. Corros. Sci..

[78] Crawford R.J., Webb H.K., Truong V.K., Hasan J., Ivanova E.P. (2012). Surface topographical factors influencing bacterial attachment. Adv. Colloid Interface Sci..

[79] Ploux L., Ponche A., Anselme K. (2010). Bacteria/Material Interfaces: Role of the Material and Cell Wall Properties. J. Adhes. Sci. Technol..

[80] Anselme K., Davidson P., Popa A.M., Giazzon M., Liley M., Ploux L. (2010). The interaction of cells and bacteria with surfaces structured at the nanometre scale. Acta Biomater..

[81] Dutta P.K., Tripathi S., Mehrotra G.K., Dutta J. (2009). Perspectives for chitosan based antimicrobial films in food applications. Food Chem..

